# Effects of recombinant Xylanase Xyn1m on growth performance, Intestinal morphology, and bacterial community structure in broilers

**DOI:** 10.1016/j.psj.2026.106961

**Published:** 2026-04-17

**Authors:** Mengjian Liu, Tingting Fu, Shaohua Zhai, Yong Chen

**Affiliations:** a Research Center for Biofeed and Animal Gut Health, College of Animal Science, Xinjiang Agricultural University, Urumqi, Xinjiang, 830052, China;; b College of Veterinary Medicine, Xinjiang Agricultural University, Urumqi, Xinjiang, 830052, China

**Keywords:** Recombinant xylanase, Xyn1m, Intestinal morphology, Gut microbiota, Broiler

## Abstract

This study evaluated the effects of recombinant xylanase Xyn1m, derived from Fibrobacter succinogenes and expressed in *Pichia pastoris*, on growth performance, intestinal morphology, and gut microbial characteristics in broilers. A total of 144 one-day-old broilers were randomly allocated to three treatments with 12 replicates per treatment (4 birds per replicate): a control group (basal diet), a low-dose Xyn1m group (6,800 IU/kg), and a high-dose Xyn1m group (13,600 IU/kg).

Supplementation with high-dose Xyn1m significantly increased body weight at 49 d and average daily gain during the 29–49 d growth phase compared with the control group (*P<* 0.01), whereas feed intake and feed conversion ratio were not affected. High-dose Xyn1m reduced duodenal crypt depth (*P<* 0.05), while ileal morphology remained unchanged. In the duodenum, Xyn1m supplementation increased the Shannon index and reduced the Simpson index of the microbial community (*P<* 0.05), although no significant differences were detected in the relative abundance of dominant bacterial taxa. Beta-diversity analysis showed that high-dose Xyn1m altered the duodenal microbial community structure. In contrast, no significant differences were observed in microbial diversity or community composition in the ileum, except for a linear reduction in the relative abundance of Acinetobacter (*P<* 0.05). Functional prediction using PICRUSt2 indicated enrichment of pathways associated with nutrient transport, carbohydrate metabolism, and energy production in the duodenal microbiota of the high-dose group, whereas predicted functional changes in the ileum were limited.

These results indicate that dietary supplementation with 13,600 IU/kg recombinant xylanase Xyn1m improved growth performance during the mid-growth stage and enhanced duodenal microbial diversity and intestinal morphology, while exerting minimal effects on distal ileal microbial functions.

## Introduction

Xylan is one of the major non-starch polysaccharides (NSP) present in cereal-based poultry feeds and is widely regarded as an anti-nutritional factor due to its complex structure and high water-binding capacity (Baker et al., 2024). It is commonly found in ingredients such as wheat, barley, corn, and soybean meal. In poultry, the presence of xylan can increase intestinal viscosity and limit the accessibility of nutrients by trapping them within plant cell walls (Wang et al., 2024). Because poultry have a relatively short gastrointestinal tract and limited endogenous xylanase activity, the digestion of these structural carbohydrates is often incomplete. As a result, nutrient utilization efficiency may be reduced, which can negatively affect growth performance and intestinal health (Wang et al., 2024).

To address these limitations, exogenous xylanases have been widely used as feed additives to improve the utilization of dietary fiber in monogastric animals. Xylanase catalyzes the hydrolysis of xylan into smaller oligosaccharides, which can reduce intestinal viscosity and improve nutrient availability. Previous studies have shown that xylanase supplementation can enhance the digestibility of dry matter and crude protein in wheat-based diets (Torres-Pitarch et al., 2019) and improve growth performance in broilers (Gao et al., 2007; Mendes et al., 2011; Santos et al., 2017). In addition to improving nutrient digestion, xylanase may also influence the intestinal microbiota by generating fermentable substrates that can be utilized by beneficial microorganisms. For example, shifts in gut microbial composition have been reported in pigs receiving dietary xylanase supplementation (Zhang et al., 2018; Hu et al., 2014). However, the effects of xylanase on the intestinal microbiota of broilers remain incompletely understood.

In our previous work, the endo-1,4-β-xylanase gene Xyn1 derived from Fibrobacter succinogenes S85 was optimized and expressed in Pichia pastoris, resulting in the recombinant enzyme Xyn1m with high enzymatic activity and favorable stability in vitro (Fu et al., 2023). Compared with many commercial xylanases, which may be susceptible to thermal degradation during feed processing or proteolytic digestion in the gastrointestinal tract, Xyn1m may offer several potential advantages. *Fibrobacter succinogenes* is a well-known cellulolytic bacterium with strong fiber-degrading capability (Béra-Maillet et al., 2004; Ransom-Jones et al., 2012). In addition, expression in the eukaryotic Pichia pastoris system enables post-translational modifications such as glycosylation, which may enhance enzyme stability and resistance to proteolytic degradation (Ahmad et al., 2014; Cereghino and Cregg, 2000). Nevertheless, the in vivo effects of this recombinant enzyme on growth performance and intestinal microbiota in broiler chickens have not yet been fully evaluated.

Therefore, the present study aimed to evaluate the effects of dietary supplementation with recombinant xylanase Xyn1m on growth performance, intestinal morphology, and gut microbial characteristics in broilers. In particular, this study focused on the potential effects of Xyn1m on microbial diversity and predicted microbial functions in different intestinal segments. The results may provide new insights into the application of recombinant xylanases for improving nutrient utilization and intestinal health in poultry production.

## Materials and methods

All experimental procedures involving animals in this study were approved by the Animal Care and Use Committee of Xinjiang Agricultural University, Urumqi, Xinjiang, China. (protocol No. 2016003)

### Preparation of recombinant xylanase Xyn1m

Recombinant xylanase Xyn1m (enzyme activity 6,800 IU/mL) was obtained by continuous high-density fermentation of the engineered yeast strain GS115-pPIC9K-Xyn1m in a 7.5 L fermenter (BioFlo®115, New Brunswick, Eppendorf, CT, USA).

Xylanase activity was determined using birchwood xylan as the substrate. The reaction mixture containing enzyme solution and 1% (w/v) birchwood xylan was incubated at 50°C in sodium citrate buffer (pH 5.3). The released reducing sugars were quantified using the dinitrosalicylic acid (DNS) method. One unit (IU) of xylanase activity was defined as the amount of enzyme required to release 1 μmoL of reducing sugars (expressed as xylose equivalents) per minute under the assay conditions.

### Birds, diets, and feeding management

A total of 144 one-day-old male Chinese yellow-feathered broilers were selected, reared on the ground until they were seven days old, and then divided into three groups at eight days of age, each with twelve replicates and four birds per replicate cage. The three groups were as follows: 1) Group C, basal diet; 2) Group L, basal diet supplemented with 6,800 IU/kg Xyn1m; and 3) Group H, basal diet supplemented with 13,600 IU/kg Xyn1m. The low dose (6,800 IU/kg) was selected as the basal effective supplementation level based on the activity of the recombinant Xyn1m preparation and our preliminary evaluation of its application in broiler diets, whereas the high dose (13,600 IU/kg) was set at two times the low dose to examine a potential dose–response relationship. On the eight-day, broilers were grouped and switched to respective diets. The basal diet was formulated based on the *Feeding Standard of Chicken* (MOA, 2004) as a reference and further optimized according to practical feeding practices, ingredient compositions, and production conditions commonly used for yellow-feathered broilers in China. The composition and nutrient levels of the diets are presented in Table 1. Prior to the trial, the chicken housing, equipment, and surrounding were disinfected with potassium permanganate solution. From the eight-day onwards, all groups were maintained on wire mesh floors with *ad libitum* access to feed and water. The experiment was performed according to the standard of chicken farm management, and the feeding experiment ended at seventy days of age.

**Table 1:** Composition and nutrient levels of basal diet (air-dry basis) %.

Ingredient (%)	1 to 28 d of age	28 to 49 d of age	49 to 70 d of age	Nutrient level 2	1 to 28 d of age	28 to 49 d of age	49 to 70 d of age
Corn	61.20	60.50	66.40	CP	20.50	19.40	16.85
Wheat bran	-	4.00	4.10	ME (MJ/kg)	12.02	12.06	12.51
Corn protein flour	3.00	3.00	4.00	OM	94.87	94.74	94.94
Soybean meal	25.00	19.40	10.90	Ca	0.70	0.80	0.83
Cottonseed protein	5.00	7.00	8.00	P	0.30	0.23	0.23
CaHPO_4_	1.30	1.00	0.90				
Premix 1	2.00	2.00	2.00				
Limestone	1.40	1.30	1.20				
Cottonseed oil	1.10	1.80	2.50				
Total	100	100	100				

1 The premix provided per kilogram of diet: VA 10,000, IU, VD₃ 32,500 IU, VE 30 IU, VK₃ 2.0 mg, VB₁ 2.2 mg, VB₂ 8.0 mg, niacin 30 mg, choline chloride 400 mg, calcium pantothenate 10 mg, VB₆ 3 mg, biotin 0.15 mg, folate 1.00 mg, VB₁₂ 0.013 mg, iron 80 mg, copper 8.0 mg, manganese 110 mg, zinc 65 mg, iodine 1.5 mg, and selenium 0.3 mg.

2 Except for metabolizable energy (ME), the nutrient levels were measured values. The ME values are approximate formulated ranges based on the original diet composition.

### Growth performance

The daily feed intake (DFI) for each replicate cage was recorded throughout the trial, and body weight (BW) was measured at 8, 28, 49, and 70 d of age. Average daily gain (ADG), average daily feed intake (ADFI), and feed-to-gain ratio (F/G) were calculated for the periods of 8–28, 29–49, and 50–70 d, as well as for the overall period of 8–70 d.

### Immunological organ index and weight measurement

At the end of the trial, one bird per replicate with live body weight (BW) closest to the cage-average BW was selected for slaughter. Immediately after euthanasia, the abdominal cavity was opened aseptically using sterile surgical instruments. The liver, spleen, thymus, and bursa of Fabricius were carefully excised, cleared of adhering connective tissue, and rinsed with ice-cold (4°C) phosphate-buffered saline (PBS, pH 7.4) to remove residual blood. Each organ was gently blotted dry with sterile filter paper and weighed to the nearest 0.01 g using an electronic balance (Mettler Toledo, Switzerland). All measurements were performed in triplicate to ensure accuracy.

The organ index (relative organ weight) was calculated as the ratio of organ weight (g) to live body weight (kg) using the Formula 1:

(1) $$\text{Organ}\;\text{index}\;\left(g/kg\right)\;=\frac{\;\text{Organ}\;\text{weight}\;\left(g\right)}{\text{Live}\;\text{body}\;\text{weight}\;\left(\text{kg}\right)}$$

### Determination of duodenal and ileum intestinal morphology

Following the removal of immunological organs, the duodenum and ileum were excised and dissected free of adjacent tissues. The total length of each segment was measured, and duodenal and ileal indices were calculated using Formula 2. A 2-cm segment was excised from both the middle part of the duodenum and ileum, and intestinal chyme was gently rinsed with 0.1 mol/L sodium phosphate buffer (pH 7.4; Waghmare et al., 2025). These segments were then fixed in Bouin's solution (for histological preservation) and stored in the dark for at least 48 h to standard histological sectioning and H.E. staining (Wu et al., 2024; Cheng, 2003). For each tissue sample, three transverse sections were prepared from the duodenal and ileal segments, respectively. Five to ten non-overlapping microscopic fields (200×magnification) per section were selected. Intestinal villus height and crypt depth were quantified using the Motic Images analysis system (Advance 3.0 software), and the villus height-to-crypt depth ratio (VCR) was calculated according to Formula 3.

(2) $$\text{The}\;\text{duodenum}\;\text{or}\;\text{ileum}\;\text{index}\;\left(\text{cm}/\text{kg}\right)=\frac{\;\text{Intestinal}\;\text{segment}\;\text{length}\;\left(\text{cm}\right)}{\text{Live}\;\text{body}\;\text{weight}\;\left(\text{kg}\right)}$$

(3) $$\text{VCR}=\frac{\text{Height}\;\text{of}\;\text{intestinal}\;\text{villi}\;\left(\mu m\right)}{\text{Depth}\;\text{of}\;\text{crypts}\;\left(\mu m\right)}$$

### Analysis of duodenum and ileal microbiota diversity, structure, and function prediction

The DNA in duodenal and ileal content was extracted using a commercial kit (Tiangen Biochemical Technology (Beijing) Co., Ltd (Beijing, China) in an aseptic environment. The DNA quality was assessed by 0.8% agarose gel electrophoresis, and the DNA purity was determined using a microplate reader (Infinite M200, Tecan, Switzerland). From each group, we randomly selected six samples of the highest quality for subsequent 16S rRNA gene amplicon sequencing. The qualified DNA served as a template for amplifying the V3-V4 region of the bacterial 16S rRNA gene using the barcoded primer pair 341F (5′-CCTACGGGGNGGCWGCAG-3′) and 806R (5′-GGACTACHVGGGGTWTCTAAT-3′) (Wu et al., 2024). The PCR products were purified and analyzed via 1×TAE at 2% agarose gel electrophoresis. Target bands were collected, and the products were purified and recovered using the GeneJET kit (Thermo Scientific, Waltham, MA, USA). DNA libraries were constructed with the NEBNext Ultra II DNA Library Prep Kit (NEB, MA, USA), quantified by Qubit, and sequenced on the Illumina MiSeq platform (Illumina Inc., San Diego, CA) by Novogene Bioinformatics Technology (Beijing, China).

The UCHIME algorithm software (https://www.drive5.com/usearch/manual/uchime_algo.html) was employed for quality control of the raw sequences. All generated clean reads were subsequently clustered using Uparse software (v 7.0, https://drive5.com/uparse/). Sequences sharing 97% similarity were grouped into the same operational taxonomic units (OTUs), with a single representative sequence selected from each OTU for taxonomic annotation via the Mothur algorithm utilizing the SILVA rRNA database (https://www.arb-silva.de/). Alpha-diversity indices of the microbiota, including OTUs and the Shannon, Simpson, and Chao1 indices, were calculated using QIIME 2 (https://qiime2.org/) (Chen et al., 2023). The beta-diversity index and principal coordinate analysis (PCoA) were assessed based on unweighted UniFrac distances. Venn diagrams illustrating the distribution of OTUs among groups were generated using https://magic.novogene.com. At the genus level, normalized relative abundance data were clustered based on Euclidean distance and visualized using the pheatmap package in R. Spearman(v 4.4.0 )correlation analysis was performed between duodenal morphology and the top 10 genera, with results plotted using corrplot. Finally, functional annotations of KEGG pathways and microbial functional predictions were performed using PICRUSt2 (v 2.6.1) and STAMP (v 3.19.2) software (Zhao et al., 2022). This approach enabled functional annotation, enrichment analysis, and prediction of significantly differentially expressed pathways across groups.

### Statistical Analysis

Statistical analyses were performed using SPSS (version 22.0, IBM Corp., Armonk, NY, USA). The cage (replicate) was considered the experimental unit. Body weight (BW) measured at 8, 28, 49, and 70 d was analyzed using a repeated-measures model with treatment, age, and their interaction included as fixed effects, cage as the experimental unit, and age specified as the repeated factor to account for temporal correlation among measurements within the same cage. Average daily gain (ADG), average daily feed intake (ADFI), and feed-to-gain ratio (F/G) were calculated for each production phase (8–28 d, 29–49 d, and 50–70 d) and analyzed by phase using the General Linear Model (GLM) procedure with treatment as a fixed effect. Orthogonal polynomial contrasts were used to evaluate dose–response effects of increasing dietary Xyn1m supplementation levels (0, 6,800, and 13,600 IU/kg), testing linear (L) and quadratic (Q) responses. When treatment effects were significant (*P* ≤ 0.05), Duncan’s multiple range test was used for pairwise comparisons. Microbial relative abundance data were analyzed using the Kruskal–Wallis non-parametric test.

Results are presented as means with SEM. Statistical significance was declared at *P* ≤ 0.05.

## Results

### Effect of Xyn1m on the growth performance

The effects of Xyn1m supplementation on the growth performance of broilers are presented in Table 2. During 8 to 28 d of age, the BW at 28 d, ADG and feed conversion efficiency linearly improved (*P* < 0.05). Compared to Group C, the BW and ADG of Groups L and H increased significantly during 29 to 49 d of age (*P* < 0.05). However, there were no significant differences among all groups in terms of ADFI (*P* > 0.05).

**Table 2:** Effect of Xyn1m on production performance of yellow broilers(g/d).

Days of age	Items	Groups			SEM	P-value		
		C	L	H		M	L	Q
8 to 28	BW at 8d	112.06	112.39	112.14	2.01	0.993	0.979	0.908
	BW at 28d	776.11	811.53	836.77	18.30	0.077	0.025	0.822
	ADG	31.62	33.31	34.53	0.81	0.052	0.016	0.813
	ADFI	47.82	47.05	48.77	1.48	0.713	0.649	0.496
	F/G	1.51	1.42	1.42	0.03	0.072	0.046	0.241
29 to 49	BW at 49d	1803.84 b	1907.50 a	1964.60 a	35.04	0.009	0.003	0.591
	ADG	48.93 b	52.20 a	53.68 a	1.04	0.009	0.003	0.490
	ADFI	113.78	114.16	119.06	2.67	0.310	0.172	0.496
	F/G	2.33	2.19	2.22	0.05	0.133	0.130	0.180
50 to 70	BW at 70d	2925.69	2990.53	3035.88	49.66	0.301	0.126	0.874
	ADG	53.40	51.56	51.03	1.33	0.428	0.218	0.691
	ADFI	158.34	152.76	158.89	4.21	0.646	0.683	0.404
	F/G	2.97	2.98	3.07	0.08	0.659	0.406	0.714
8 to 70	ADG	44.66	45.68	46.41	0.77	0.285	0.118	0.874
	ADFI	106.64	104.65	107.91	2.26	0.596	0.696	0.351
	F/G	2.39	2.29	2.33	0.05	0.325	0.331	0.253

Abbreviations: BW, body weight (g); ADG, average daily gain; ADFI, average daily feed intake; F/G, ratio of ADFI to ADG. M, main effect of treatment; L, linear effect; Q, quadratic contrast.

a-b Mean values without a common superscript letter within a row are significantly different (*P* < 0.05).

### Effect of Xyn1m on immunological organs

As shown in Table 3, no significant differences were observed in the immunological organ indices or weights among the groups (*P* > 0.05).

**Table 3:** Effect of Xyn1m on immunological organs of yellow broilers.

Items	Groups			SEM	P-value			
	C	L	H		M	L	Q	
Liver index (g/kg)	1.70	1.87	1.65	0.07	0.408	0.746		0.196
Spleen index (g/kg)	0.17	0.15	0.15	0.00	0.362	0.192	0.574	
Thymus index (g/kg)	0.40	0.35	0.37	0.02	0.709	0.634	0.501	
Bursa index (g/kg)	0.18	0.17	0.15	0.01	0.530	0.266	0.903	
Liver weight (g)	47.77	56.96	49.39	2.25	0.211	0.768	0.084	
Spleen weight (g)	4.68	4.56	4.43	0.19	0.865	0.593	0.980	
Thymus weight (g)	11.18	10.57	11.19	0.59	0.891	0.997	0.633	
Bursa weight (g)	5.07	4.96	4.60	0.27	0.757	0.480	0.823	

### Effect of Xyn1m on duodenal and Ileal morphology

As shown in Tables 4, morphological assessments of the duodenum and ileum revealed segment-specific responses to Xyn1m supplementation. In the duodenum, Group L exhibited significantly longer duodenal length than Group C (*P* < 0.05), accompanied by a main effect (M, *P* = 0.027) and quadratic effect (Q, *P* = 0.042). Additionally, Group H significantly reduced crypt depth compared to Group C (*P* < 0.05), with a significant main effect (M, *P* = 0.045) and linear effect (L, *P* = 0.019). These results indicate that Xyn1m primarily modulates proximal gut (duodenal) morphology, with minimal impact on distal (ileal) structural characteristics.

**Table 4:** Effect of Xyn1m on gut morphology of yellow broilers.

Intestinal segment	Item	Groups			SEM	P-value		
		C	L	H		M	L	Q
Duodenum	Length (cm)	24.67^b^	27.63^a^	26.68a^b^	0.75	0.027	0.066	0.042
	Duodenal index (cm/kg)	8.81	9.13	8.92	0.27	0.689	0.774	0.419
	Villus height (μm)	1423.10	1433.04	1430.11	23.21	0.953	0.832	0.822
	Crypt depth (μm)	199.07^a^	180.29^ab^	175.97^b^	6.64	0.045	0.019	0.381
	VCR	7.28	8.01	8.28	0.322	0.092	0.036	0.554
Ileum	Length (cm)	64.13	66.89	64.21	1.03	0.286	0.974	0.300
	ileum index (cm/kg)	22.98	22.09	21.47	0.40	0.370	0.132	0.530
	Villus height (μm)	788.46	846.45	813.63	14.22	0.101	0.470	0.347
	Crypt depth (μm)	129.56	130.88	125.69	1.83	0.772	0.397	0.259
	VCR	6.12	6.51	6.50	0.13	0.212	0.231	0.960

Abbreviations: VCR, villus height-to-crypt depth ratio.

a-b Mean values without a common superscript letter within a row are significantly different (*P* < 0.05).

### The gut microbiota

In this experiment, the V3-V4 region of 16S rDNA in duodenal and ileal content samples from broiler chickens were analyzed using the Illumina MiSeq sequencing platform. A total of 1,418,236 and 1,228,662 valid tags were obtained from the sequencing of the duodenum and ileum of 18 samples, respectively. Following high-throughput sequencing and quality control, an average of over 78,000 and 68,000 clean reads of bacterial 16S rDNA were acquired from each sample.

### Alpha diversity analyses

The effects of Xyn1m on the microbial α-diversity indices of chicken intestinal bacteria are presented in Table 5. Compared to Group C, the Shannon index of duodenal bacteria for both Group H and Group L was significantly increased (*P* < 0.05). However, the Simpson index for Group L was significantly decreased (*P* < 0.05). These findings indicate that Xyn1m significantly enhances the diversity and evenness of the duodenal microbiota, while also contributing to an increase in microbiota richness to a certain extent.

**Table 5:** Effects of Xyn1m on alpha diversity index of intestinal microflora.

Intestinal segment	Item	Groups			SEM	P-value		
		C	L	H		M	L	Q
Duodenum	OUTs	394.17	432.67	408.33	29.21	0.769	0.429	0.615
	Shannon	2.84 b	3.71 a	3.53 a	0.32	0.015	0.044	0.588
	Simpson	0.80 a	0.62 b	0.74 ab	0.04	0.035	0.131	0.479
	Chao 1	431.13	467.37	440.07	29.23	0.790	0.420	0.288
	ACE	434.43	471.54	444.73	27.56	0.890	0.720	0.620
Ileum	OTUs	336.67	301.00	279.33	29.21	0.640	0.455	0.776
	Shannon	3.58	3.19	2.95	0.32	0.635	0.446	0.769
	Simpson	0.80	0.71	0.68	0.04	0.398	0.253	0.755
	Chao1	366.98	327.40	308.13	29.23	0.604	0.443	0.800
	ACE	370.28	343.43	317.60	27.56	0.709	0.467	0.720

Abbreviations: OTUs, operational taxonomic units; ACE, abundance-based coverage estimator.

a-b Mean values without a common superscript letter within a row are significantly different (*P* < 0.05).

### Beta diversity analyses

**Fig. 1:**
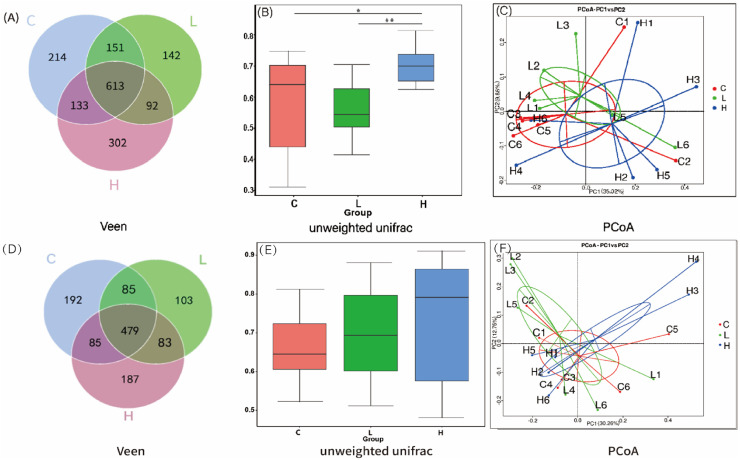
Analysis of duodenal and ileum bacterial OTUs and β-diversity. Venn diagrams of OTUs in duodenal (A) and ileal (D) bacteria across different treatment groups; Comparisons of β-diversity indices for duodenal (B) and ileal (E) bacteria, calculated using unweighted UniFrac distances; Principal coordinate analysis (PCoA) of duodenal (C) and ileal (F) bacterial communities at the OTU level, based on unweighted UniFrac distances. In duodenal: C, the control group; L, the low-dose group; H, the high-dose group. In ileum: C, the control group; L, the low-dose group; H, the high-dose group.*, *P* < 0.05; **, *P* < 0.01.

The results of the duodenal bacterial OTUs and β-diversity are presented in Fig. 1A, 1B and 1C. A total of 613 OTUs were shared among the three groups, while 214, 142, and 302 unique OTUs were identified in Group C (control), Group L (low-dose), and Group H (high-dose), respectively (Fig. 1A). These findings indicate that the Xyn1m supplementation influenced the microbial community structure of the duodenal contents in broiler. According to the unweighted UniFrac distance, the β-diversity index was significantly higher in Group H compared to Group C (*P* < 0.05) and Group L (*P* < 0.01) (Fig. 1B). The principal coordinate analysis (PCoA) at the OTUs level revealed that PC1 accounted for 35.02% and PC2 for 9.86% of the variance in bacterial communities (Fig. 1C). Group C was predominantly located in the middle-left region, Group L in the upper left region, and Group H in the bottom right region. The three groups were distinctly concentrated and could be differentiated, indicating that both low and high doses of Xyn1m altered the microbial community structure in the duodenum of yellow-feathered broilers.

The ileal bacterial OTUs and β-diversity are given in Fig. 1D, 1E and 1F. Venn diagram revealed that 479 shared OTUs across the three groups. Specifically, 192, 103, and 187 unique OTUs were identified in Groups C (control), L (low-dose), and H (high-dose), respectively (Fig. 1D). While the β-diversity index was highest in Group H, no significant differences were detected in the ileal microflora among groups (Fig. 1E). The PCoA at the OTUs level showed that PC1 and PC2 explained 30.26% and 12.75% of the variance in bacterial community structures, respectively (Fig. 1F). However, the microbial communities of Groups C, L, and H did not exhibit significant clustering or differences, indicating that Xyn1m had no effect on the structural composition of the ileal microbial community.

### Relative abundances of MetaStats analysis

**Fig. 2:**
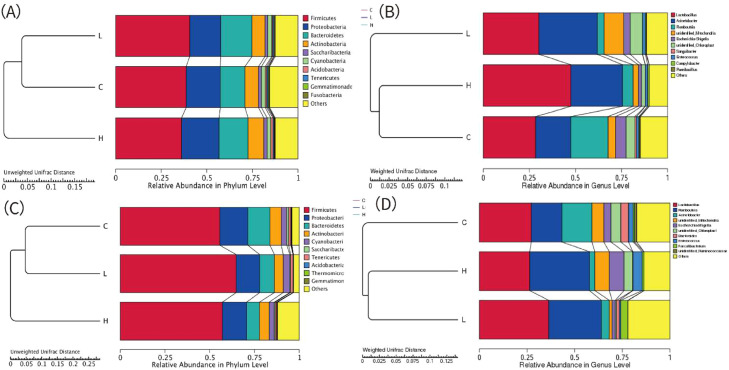
Relative abundances of broiler duodenal and ileum bacteria and MetaStats analysis of the differences at the phylum (A and C), genus levels (B and D). In duodenal: C, the control group; L, the low-dose group; H, the high-dose group. In ileum: C, the control group; L, the low-dose group; H, the high-dose group.

As shown in Fig. 2A and 2C, at the phylum level, the dominant bacterial phyla in both the duodenum and ileum included *Firmicutes, Proteobacteria, Bacteroidetes,* and *Actinobacteria*. At the genus level (Fig. 2B and 2D), compared to the control group, the high-dose Xyn1m group numerically increased relative abundance of *Lactobacillus* alongside decreased abundances of *Romboutsia* and *Escherichia-Shigella* in duodenum.

### Analysis of differences in relative abundance of species

The effects of Xyn1m on the duodenal and ileal microbiota of broilers at the phylum and genus levels are summarized in Table 6. Only core microbial taxa (top 10) were selected as the focus of this study, specifically those exhibiting significant differences in abundance, or those with a relative abundance more than 1% accompanied by directional changes and meaningful statistical signals.

**Table 6:** Effects of Xyn1m on intestinal microflora.

Intestinal segment	Items	Groups			SEM	P-value		
		C	L	H		M	L	Q
Duodenum	Firmicutes	40.93	55.85	57.95	26.90	0.518	0.299	0.647
	Proteobacteria	47.78	32.18	35.04	24.00	0.517	0.382	0.463
	Lactobacillus	28.32	30.22	47.50	10.92	0.524	0.309	0.633
	Acinetobacter	19.16	31.62	27.97	25.33	0.707	0.571	0.550
	Romboutsia	20.20	3.71	5.78	15.96	0.150	0.116	0.234
	Escherichia_Shigella	5.72	3.60	1.70	1.13	0.413	0.103	0.959
Ileum	Firmicutes	53.82	88.38	70.36	22.85	0.121	0.307	0.071
	Proteobacteria	33.42	7.97	20.98	14.89	0.102	0.277	0.062
	Lactobacillus	27.31	36.35	26.41	12.17	0.846	0.962	0.570
	Acinetobacter	15.83^a^	4.23 ^b^	2.76 ^b^	5.99	0.043	0.022	0.272
	Romboutsia	15.96	27.61	31.42	8.10	0.555	0.305	0.760

In the duodenum, the relative abundances of the dominant phyla and genera did not show any statistically significant differences across the treatment groups (*P* > 0.05). In the ileum, Acinetobacter was the only genus that exhibited a statistically significant linear decrease in relative abundance with Xyn1m supplementation (*P* *<* 0.05). No significant differences were observed in the relative abundances of other dominant ileal phyla or genera across the groups (*P* > 0.05).

### Relative abundance cluster heatmap analysis at the genus level

**Fig. 3:**
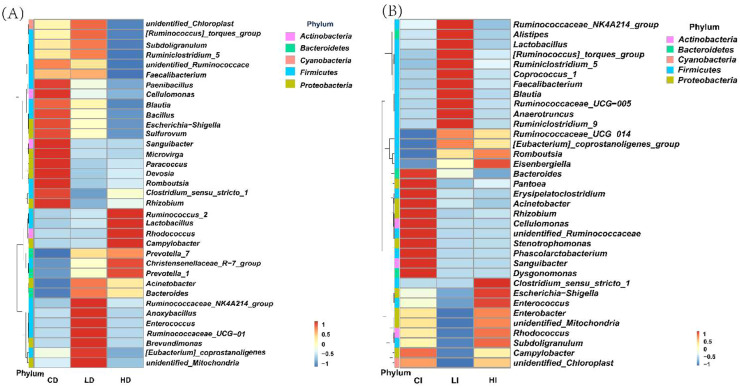
Relative abundance cluster heatmap analysis at phylum level in duodenal (A) and ileum (B).

As shown in Fig. 3A, the heatmap of relative abundance clustering revealed clear differences in microbial composition among the three groups in the duodenum, and a clear difference appeared in 3 groups. Group C clustering mainly in *Paenibacillus, Cellulomonas, Blautia, Bacillus, Escherichia- Shigella, Sulfurovum, Sanguibacter, Microvirga, Paracoccus, Devosia, Romboutsia, Clostridium-sensu-stricto-1* and *Rhizobium*; Group L clustered mainly in the *Ruminococcaceae_NK4A214_group, Anoxybacillus, Enterococcus, Ruminococcaccac_UCG-014, Brevundimonas, Coprostanoligenes,* and *unidentified Mitochondria*; Group H mainly clusters with *Ruminococcus_2, Lactobacillus, Rhodococcus,* and *Campylobacter, Prevoiclla_7, Christensenellaceae_R-7_groupand Prevolella_1.*

As shown in Fig. 3B, the heatmap of ileal microbial relative abundance indicated distinct clustering patterns among the treatment groups. Group C clustering mainly in *Bacteroides, Pantoea, Erysipelatoclostridium, Acinetobacter, Rhizobium, Cellulomonas, unidentified Ruminococcaceae, Stenotrophomonas Phascolarctobacterium,* and *Sanguibacter, Dysgonomonas;* Group L clustered mainly in the *Ruminococcaceae NK4A214_group, Alistipes, Lactobacillus, Ruminococcus torques_group, Ruminiclostridium_5, Coprococcus_1, Faecalibacterium, Blautia, Ruminococcaceae UCG-005, Anaerotruncus, Ruminiclostridium_9;* Group H mainly clusters with *Clostridium sensu_stricto_1, Escherichia-Shigella, Enterococcus, Enterobacter, unidentified Mitochondria, Rhodococcus, Subdoligranulum.*

### Correlation analysis between duodenal morphology and top 10 duodenal bacterial genera

**Fig. 4:**
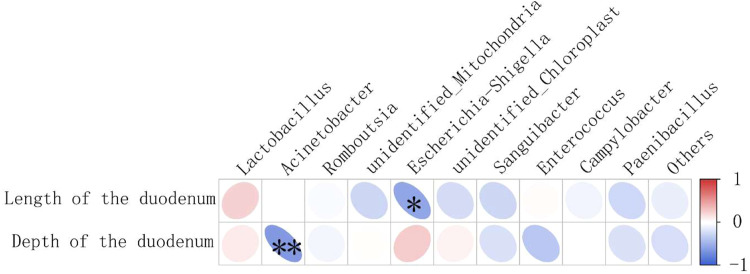
Correlation Heatmap between morphology and top 10 microflora of duodenum at genus level.

The relationships between duodenal morphology and the relative abundance of the dominant genera are presented in Fig. 4. To elucidate the interplay between duodenal morphology and microbial composition, Spearman correlation analysis was performed to assess the relationships between morphological parameters with significant differences among groups (length and crypt depth of duodenum) and the relative abundances of the top 10 most abundant in the duodenum (*Lactobacillus, Acinetobacter, Romboutsia, unidentified_Mitochondria, Escherichia-Shigella, unidentified_Chloroplast, Sanguibacter, Enterococcus, Campylobacter, Paenibacillus*).

A significant negative correlation was observed between *Acinetobacter* abundance and crypt depth (*P* < 0.01), suggesting that higher *Acinetobacter* levels were associated with shallower crypts, indicative of reduced epithelial cell proliferation. Conversely, *Escherichia-Shigella* exhibited a significant negative correlation with duodenal length (*P* < 0.05), implying that elevated *Escherichia-Shigella* abundance corresponded to shorter duodenal dimensions, potentially reflecting impaired intestinal development.

### Functional prediction of duodenal and ileal microbiota

**Fig. 5:**
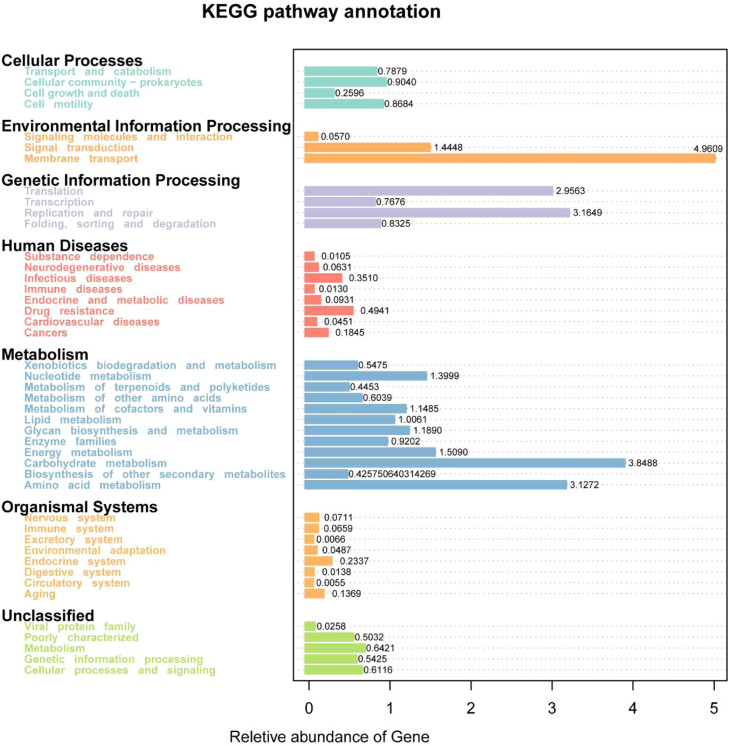
KEGG pathway annotation of microbiota in duodenum.

**Fig. 6:**
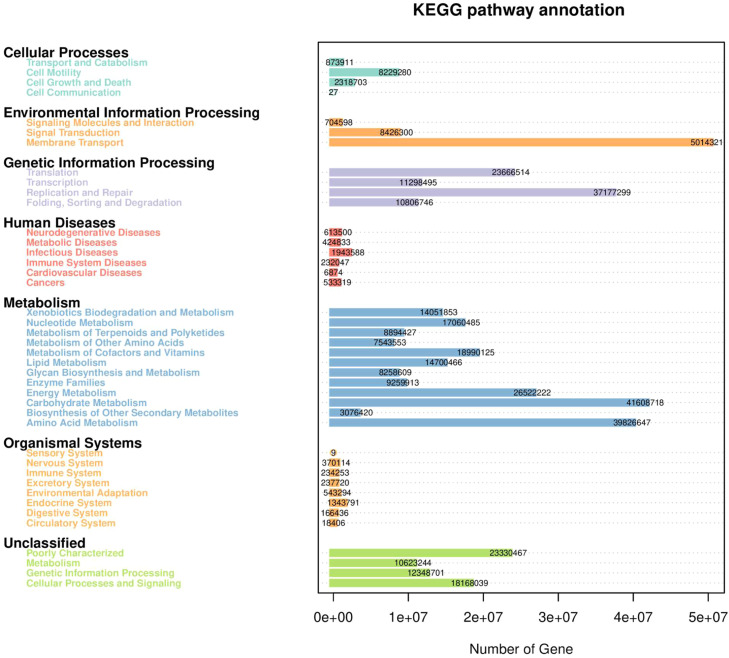
KEGG pathway annotation of microbiota in ileum.

**Fig. 7:**
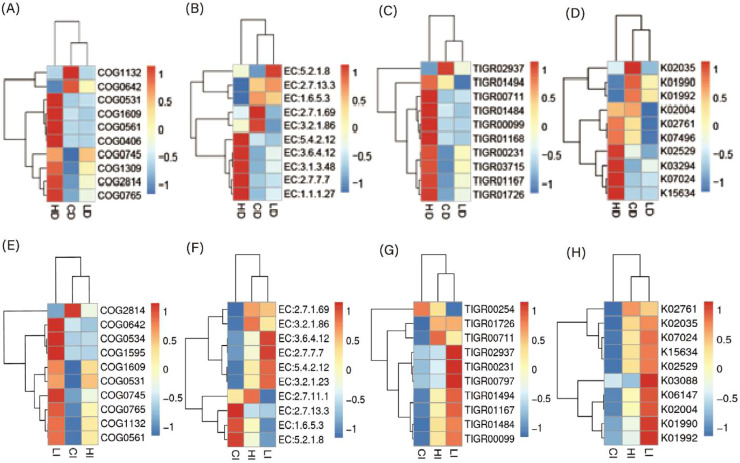
Heatmap analysis of microbial functional predictions in duodenal (A-D) and ileum (E-H). Clusters of Orthologous Groups (COG) (A and E); Enzyme Commission (EC) (B and F);TIGRFAM (TIGR Families of protein domains) (C and G); KEGG Ortholog (KO) (D and H).

**Fig. 8:**
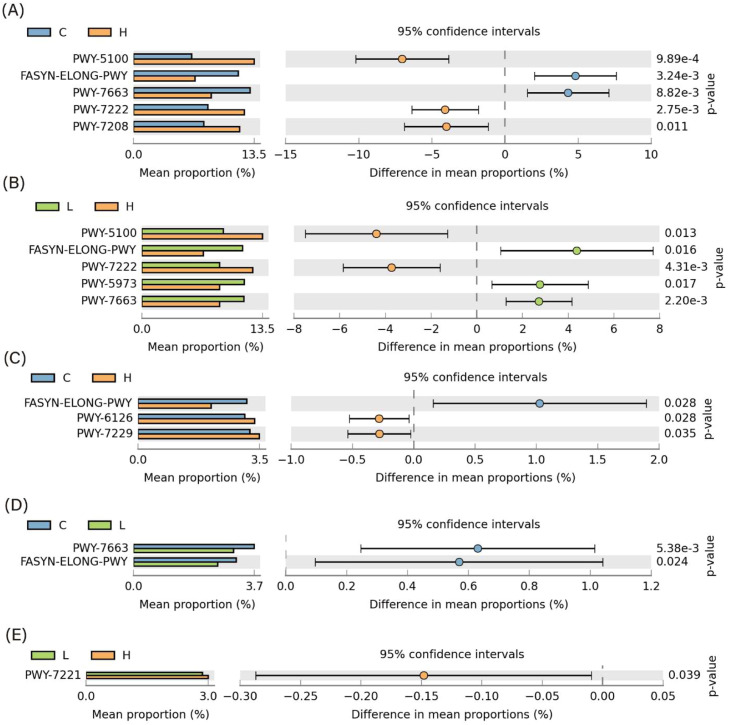
Differential analysis of enriched T-test in predicting microbial pathways in duodenal (A and B) and ileum (C-E). Group definitions: C, control group; L, low-dose group; H, high-dose group.

Functional prediction analysis using PICRUSt2 and STAMP is presented in Figs. 5–8. As shown in Fig. 5, several predicted metabolic pathways were enriched in the duodenal microbiota of the high-dose Xyn1m group. Fig. 5 illustrates significant enrichment of nutrient transport pathways (amino acid/sugar transporters), glucose metabolism (lactate dehydrogenase), and energy conversion (ATP synthase) in H duodenal microbiota. Notably, pyruvate fermentation pathways (PWY-7222, PWY-5100) and pyrimidine salvage (PWY-7208) were upregulated (*P* < 0.05), while anaerobic gonadotropin biosynthesis and saturated fatty acid elongation pathways were downregulated, as shown in Fig. 8A-B—findings consistent with enhanced nutrient utilization. Fig. 7A-D demonstrates that H duodenal functions shifted toward amino acid uptake (COG0531), anaerobic fermentation (EC:1.1.1.27), and energy production (EC:3.6.4.12), with enrichment of genes such as sucrose-6-phosphatase (K07024) and phosphoglycerate mutase (K15634).

In contrast, as shown in Fig. 6, the predicted functional profiles of the ileal microbiota showed only limited differences among groups. With only subtle trends in ATP synthesis (EC:3.6.4.12) and stress resistance (TIGR00711) observed in Xyn1m-treated groups, which shown in Fig. 7E-H. The differential pathway analysis is presented in Fig. 8A–E. further showed that ileal fatty acid elongation pathways were downregulated in the low-dose Xyn1m group, whereas high-dose Xyn1m additionally suppressed gonadotropin biosynthesis.

As visualized in Fig. 5–8, these results indicate that Xyn1m selectively optimizes proximal gut metabolic pathways to enhance nutrient absorption, supporting its role in improving broiler growth performance with limited impact on distal ileal functional profiles.

## Discussion

### Effect of Xyn1m on the growth performance of broilers

The improvement in growth performance observed in the present study is likely associated with the ability of Xyn1m to degrade dietary non-starch polysaccharides (NSP). Hydrolysis of these structural carbohydrates reduces intestinal viscosity and facilitates the release of nutrients trapped within plant cell walls, thereby improving the accessibility of endogenous digestive enzymes (Kaczmarek et al., 2014; Li et al., 2019; Ma et al., 2009). In addition, the breakdown of xylan generates fermentable oligosaccharides that may serve as substrates for beneficial intestinal microorganisms. These combined effects can improve nutrient digestion and absorption, which may ultimately support better growth performance in broilers.

In the present study, supplementation with 13,600 IU/kg Xyn1m increased body weight at 49 d and average daily gain during the 29–49 d period. This suggests that the beneficial effects of the enzyme were most evident during the rapid growth phase. During this stage, broilers exhibit increased nutrient requirements, and improvements in nutrient availability may have a greater impact on growth performance.

Feed intake did not differ among treatments, indicating that the observed growth improvement was not associated with increased feed consumption but rather with improved nutrient utilization. One possible explanation is that xylanase disrupts the “cage effect” of plant cell walls, thereby increasing the availability of nutrients encapsulated within the feed matrix (Bedford and Cowieson, 2012). In addition, the intestinal microbiota of older broilers is generally more stable and metabolically active, which may allow more efficient utilization of xylo-oligosaccharides produced by xylanase activity (Bautil et al., 2019). These factors together may explain why the growth response was most pronounced during the mid-growth stage.

### Effect of Xyn1m on immunological organs

No significant differences were observed in the relative weights of immune organs among treatments, suggesting that Xyn1m supplementation did not influence systemic immune organ development. Similar observations have been reported in previous studies where xylanase supplementation improved intestinal health without altering the size of major lymphoid organs (Wang et al., 2024).

Although systemic immune indices remained unchanged, the changes observed in the intestinal microbiota may still contribute to local immune regulation. In particular, the increase in *Lactobacillus* and the reduction in *Escherichia-Shigella* in the duodenum may help maintain intestinal immune homeostasis. *Lactobacillus* species are widely recognized for their ability to inhibit pathogenic bacteria through competitive exclusion and the production of organic acids (Callaghan et al., 2021). Therefore, the maintenance of stable immune organ indices may reflect a balanced intestinal environment rather than a stimulation of systemic immune activity (Daneshmand et al., 2023).

### Effect of Xyn1m on duodenal and Ileal morphology

In the present study, Xyn1m supplementation reduced crypt depth in the duodenum, while no significant changes were observed in the ileum. A reduction in crypt depth is generally considered indicative of lower epithelial turnover and improved intestinal stability. Previous studies have also reported that dietary xylanase can improve intestinal morphology by reducing intestinal irritation and enhancing nutrient digestion (Cheng et al., 2016; Ding et al., 2010).

The localized effect observed in the duodenum may be related to the site of enzymatic activity. Xylanase primarily acts on dietary fiber shortly after ingestion, which means that the proximal intestine is more likely to experience changes in substrate availability and microbial fermentation. The enrichment of *Lactobacillus* observed in the duodenum may further support mucosal integrity, as *Lactobacillus* species are known to contribute to intestinal barrier maintenance and to reduce inflammatory responses in the gut (Callaghan et al., 2021).

In contrast, the absence of morphological changes in the ileum may be explained by the depletion of fermentable substrates before the digesta reaches the distal intestine. Extensive hydrolysis of dietary xylan in the upper gastrointestinal tract likely reduces the amount of residual fiber available for fermentation in the ileum. Consequently, both microbial composition and intestinal morphology in the distal gut remained relatively stable. Similar segment-specific responses have been reported in previous studies evaluating dietary xylanase supplementation (Mathlouthi et al., 2002; Zhang et al., 2018).

### The Effect of Xyn1m on the relative abundance and diversity of gut microbiota

Dietary xylanase can influence intestinal microbiota by modifying the availability of fermentable substrates derived from dietary fiber. In poultry, which possess a relatively short gastrointestinal tract and limited endogenous fiber-degrading enzymes, these changes may be particularly important (Daneshmand et al., 2023). By hydrolyzing complex xylan structures, Xyn1m may alter the metabolic environment of the intestinal lumen, thereby influencing microbial diversity and community composition.

In the present study, Xyn1m supplementation increased the Shannon index of the duodenal microbiota, suggesting greater microbial diversity in this intestinal segment. Increased microbial diversity is often considered a characteristic of a more stable intestinal ecosystem.

In contrast, the microbial diversity and community structure of the ileum remained largely unchanged. This difference between intestinal segments likely reflects the spatial variation in substrate availability along the gastrointestinal tract. Because a substantial portion of dietary xylan may already be hydrolyzed in the proximal intestine, the distal gut receives fewer fermentable substrates, which may limit further microbial shifts in the ileum. These results are consistent with previous observations that xylanase supplementation tends to exert stronger effects in the upper gastrointestinal tract than in distal segments (Zhang et al., 2018).

### Effect of Xyn1m on the microbiota characteristics

Although no statistically significant differences were detected in the relative abundance of dominant taxa in the duodenum, several numerical trends were observed. In particular, Xyn1m supplementation was associated with increased relative abundance of *Lactobacillus* and reduced abundance of *Escherichia-Shigella*. These trends may still be biologically relevant because *Lactobacillus* species are generally considered beneficial members of the intestinal microbiota and play important roles in maintaining gut health through the production of organic acids and inhibition of pathogenic bacteria (Callaghan et al., 2021).

Conversely, *Escherichia-Shigella* and *Acinetobacter* include opportunistic pathogens that may contribute to intestinal dysfunction under certain conditions (Baltazar-Díaz et al., 2022; Wang et al., 2021). Therefore, even modest reductions in these taxa may help maintain intestinal microbial balance. Similar shifts toward beneficial microbial communities have been reported in broilers receiving xylanase-treated or fermented feed ingredients (Feng et al., 2020).

Importantly, these microbial changes were primarily observed in the duodenum, whereas the ileal microbiota remained relatively stable. This regional difference supports the idea that the ecological effects of xylanase are concentrated in the proximal intestine where enzymatic hydrolysis of dietary fiber occurs.

### Effect of Xyn1m on microbial functions prediction

Functional prediction analysis based on PICRUSt2 suggested potential differences in microbial metabolic pathways in response to Xyn1m supplementation. It should be noted that these results represent predicted functional potentials derived from 16S rRNA gene data rather than direct measurements of microbial metabolic activity.

In the duodenum, predicted pathways associated with nutrient transport, carbohydrate metabolism, and energy production were relatively enriched in the high-dose group. These functional predictions may reflect increased microbial utilization of oligosaccharides produced during xylan hydrolysis. The enrichment of pathways related to pyruvate fermentation and short-chain fatty acid production may also contribute to improved intestinal function and nutrient utilization (Callaghan et al., 2021).

In contrast, predicted functional differences in the ileal microbiota were limited. This observation is consistent with the relatively stable microbial composition observed in the ileum and further supports the idea that the primary effects of Xyn1m occur in the proximal intestinal segments.

## Conclusions

Dietary supplementation with recombinant xylanase Xyn1m at 13,600 IU/kg improved growth performance during the mid-growth phase (29-49 days) and enhanced duodenal microbial diversity and potential functional activity and intestinal morphology. However, no significant effects were observed on overall growth performance across the entire experimental period. These findings indicate that Xyn1m may exert stage-specific benefits by modulating proximal intestinal environments.

## CRediT authorship contribution statement

**Mengjian Liu:** Writing – original draft, Visualization, Formal analysis, Data curation. **Tingting Fu:** Methodology. **Shaohua Zhai:** Investigation, Data curation. **Yong Chen:** Writing – review & editing, Supervision, Project administration.

## Disclosures

The authors declare no conflicts of interest.
